# Numerical Investigation of Vortex-Induced Enhancement in the Mixing Characteristics of Double-Spiral and Serpentine Microchannels

**DOI:** 10.3390/mi16091016

**Published:** 2025-08-31

**Authors:** Litao Qin, Zhen Jiang, Dongjian Zhou, Jincai Yue, Huanong Cheng

**Affiliations:** College of Chemical Engineering, Qingdao University of Science and Technology, Qingdao 266042, China; qin_lt@163.com (L.Q.); chn@qust.edu.cn (H.C.)

**Keywords:** double-spiral microchannel, serpentine microchannel, groove structure, enhancing mixing, numerical simulation

## Abstract

To enhance passive mixing in microchannels, T-shaped double-spiral and serpentine microchannels with identical curvature radii were designed and numerically analyzed across a Reynolds number (*Re*) range of 1 to 300. The double-spiral microchannel exhibited superior mixing performance at *Re* ≤ 200, which is primarily attributed to the efficient utilization of Dean vortices. In contrast, the serpentine microchannel showed better performance at *Re* ≥ 250, benefiting from the early formation of four-vortex structures induced by periodic curvature reversals. To further enhance the performance of the serpentine microchannel at low *Re*, groove structures with varying orientation angles were incorporated. The introduction of the groove structures generated lateral secondary flows that not only increased flow disturbances but also disrupted the symmetry of the Dean vortices. Among these configurations, Structure 2, with a 45° angle between the groove direction and centrifugal force, exhibited the most pronounced enhancement in vortex intensity, as the secondary flows induced by the grooves synergistically interacted with the Dean vortices. This configuration resulted in the highest mixing enhancement (>50%). This study provides valuable insights into geometry-driven mixing mechanisms and offers design guidelines for high-efficiency micromixers across a wide range of *Re*.

## 1. Introduction

Microchannels have attracted considerable attention in chemical engineering applications due to their unique advantages. The fluid flow within these channels is predominantly laminar, owing to their extremely small dimensions (hydraulic diameter less than 1 mm). As a result, mixing in microchannels is primarily controlled by molecular diffusion and chaotic advection [1,2,3], which leads to inherently poor mixing performance. To overcome this limitation, numerous studies have focused on designing microchannel structures that enhance fluid flow disturbances, induce secondary flows or vortices, and increase the contact surface area, thereby improving mixing efficiency [4,5]. The optimization and improvement of geometric structures continue to be a key focus in this field of research.

The planar curved microchannel utilizes centrifugal and shear forces to generate Dean vortices, which enhance fluid mixing [6,7,8,9,10] and improve heat transfer [11,12,13]. Commonly utilized curved microchannel geometries, such as spiral [14,15,16,17,18,19] and serpentine [20,21,22,23] structures, are characterized by their simple design, low pressure drop, high mixing efficiency, and uniform residence time, making them particularly suitable for high Reynolds number (*Re*) mixing applications. Tripathi et al. [18] conducted numerical simulations to investigate the mixing performance of straight, wavy, and spiral microchannels across a *Re* range of 1 to 100. Their findings revealed that, for the same channel length, spiral microchannels exhibited the best mixing performance, followed by wavy microchannels, with straight microchannels showing the poorest performance. However, their study did not explore the influence of curvature radius on mixing performance, a factor that differs between spiral and wavy microchannels. Wang et al. [20] introduced an elliptical serpentine microchannel design and demonstrated that Dean vortex strength is influenced by the curvature of the ellipse. Their simulations indicated that higher eccentricity leads to stronger Dean vortices, thereby enhancing mixing performance. However, it is essential to note that the eccentricity of the ellipse cannot be increased indefinitely. Zhou et al. [21] compared the mixing characteristics of spiral and serpentine microchannels across a wide *Re* range (1–500) through both numerical simulations and experiments. Their results showed that the mixing performance of both designs exhibited distinct behaviors based on *Re*, which were attributed to vortex evolution. Although Zhou’s study primarily focused on single-spiral microchannels, practical applications often require the serial connection of multiple mixing units. In this context, the double-spiral structure offers advantages over the single-spiral design, as it facilitates easier integration and arrangement of multiple mixing units.

The intensity of Dean vortices in curved microchannels is highly sensitive to the *Re*. Specifically, at low *Re*, the weakened Dean vortices lead to significantly reduced flow perturbations, which in turn compromise the mixing efficiency. To overcome this limitation, various studies have explored strategies to enhance the mixing performance of curved microchannels at low *Re*, including the introduction of obstacles such as circular [24,25] and hexagonal prisms [26,27], as well as triangular or teardrop-shaped baffles [28,29,30]. While these modifications enhance mixing, they also contribute to an increase in fluid flow resistance, resulting in higher pressure drops. For instance, Corning’s Advanced-Flow Reactors employ baffles and cylindrical obstacles within their heart-shaped chambers, significantly increasing the resistance of the microreactor. However, this comes at the cost of more complex structural designs and greater fabrication challenges [31]. An alternative and effective strategy for improving mixing in curved microchannels involves the incorporation of ridges [32,33] or grooves [34,35,36,37,38,39,40] on one or more of the channel walls. Alam et al. [34] investigated the mixing performance of a serpentine microchannel with grooves on the side walls and demonstrated that the grooved microchannel achieved a 135% higher mixing index compared to its smooth counterpart. Similarly, Rhoades et al. [36] integrated herringbone grooves into serpentine microchannels, enhancing mixing through the synergistic interaction between Dean vortices and helical flows induced by the inclined grooves. Hadj et al. [40] systematically explored the influence of groove structural parameters on mixing performance in serpentine microchannels, although their study was limited to an exceptionally low *Re* range of 0.3–5, corresponding to creeping flow conditions. The findings of these studies indicate that grooves can significantly enhance mixing performance without causing additional energy consumption. In contrast to obstacles, the design and fabrication of microchannels with grooves are simpler and more efficient. Although these studies have indicated that grooves can enhance mixing in serpentine microchannels, most of the research has focused on a relatively small Reynolds number range, typically below 100. At such low Re, the Dean vortex structure is simple, and the coupling mechanism between the grooves and multi-vortex structures has not been explored.

The Dean number in curved microchannels significantly influences both flow and mixing characteristics. Although extensive research has underscored the significant interest in spiral or serpentine microchannels, most studies have examined these structures in isolation or compared spiral and serpentine microchannels with different curvature radii, often overlooking the variations in their respective Dean numbers. This limitation makes direct comparisons between these configurations less convincing. Zhou et al. [21] conducted a comparative study between single-spiral and serpentine microchannels but did not address the practically more relevant double-spiral structure. In this study, the mixing characteristics of the double-spiral microchannel and its expanded serpentine counterpart are investigated, with a particular focus on the influence of Dean vortices on mixing performance across the *Re* range of 1 to 300, analyzed through numerical simulation. Given the relatively poor mixing performance of the serpentine microchannel, grooves are introduced to enhance mixing efficiency. The synergistic effects of Dean vortices and secondary flows induced by the grooves are explored over a wide range of *Re*, and the mixing performance of serpentine microchannels with varying groove structural angles is evaluated. This study contributes to a comprehensive understanding of the fundamental reasons behind the flow pattern and mixing characteristic differences caused by the shape of various curved channels. It also enhances the understanding of the mechanisms of Dean vortex-enhanced mixing in curved channels and deepens the insight into the groove-enhanced mixing effect.

## 2. Numerical Method

### 2.1. Geometric Model

**Figure 1:**
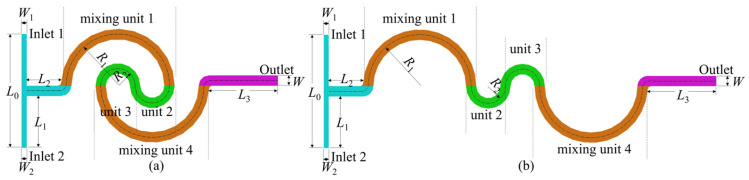
Schematic diagrams of the microchannels: (**a**) double-spiral and (**b**) serpentine.

The planar schematic diagrams of the T-shaped double-spiral and serpentine microchannels are presented in Figure 1. The double-spiral microchannel consists of four semi-circular sections, each functioning as a distinct mixing unit. The serpentine microchannel is an extension of the double-spiral microchannel. In both configurations, sections marked with the same color represent identical structures and channel lengths, ensuring uniform curvature radii across the two microchannels. Each microchannel features two inlets and one outlet. The inlet cross-section is rectangular, with dimensions of 150 × 300 μm (width × depth), whereas both the channel cross-sections and the outlet are square, measuring 300 × 300 μm. The detailed geometric parameters are summarized in Table 1.

**Figure 2:**
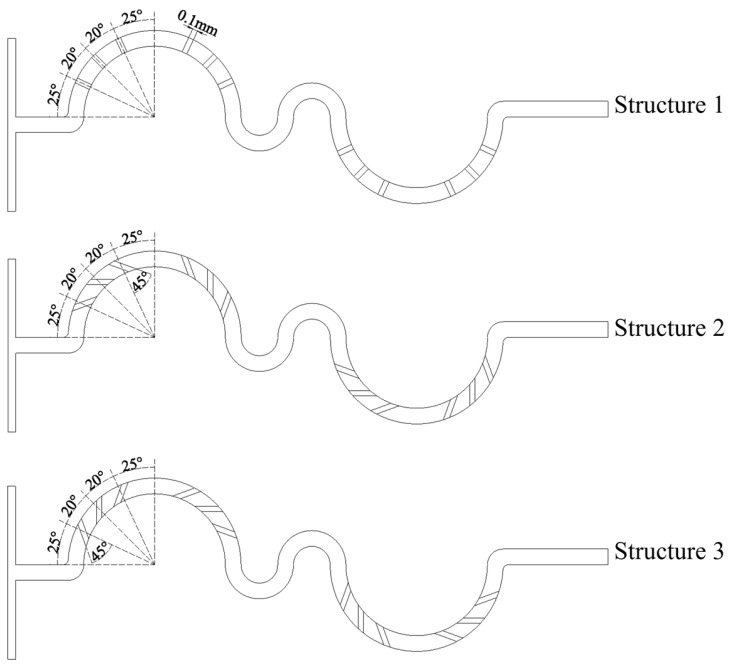
Schematic diagrams of serpentine microchannel with grooves.

**Figure 3:**
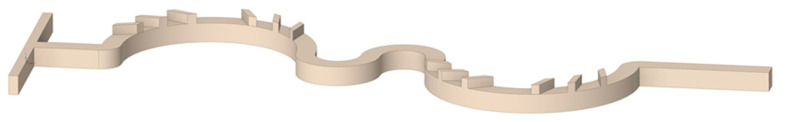
Three-dimensional schematic diagram of Structure 2.

The serpentine microchannel is modified by incorporating groove structures, each with a width of 100 μm and a depth of 150 μm, as shown in Figure 2. The grooves are positioned within the first and fourth mixing units, with a total of 12 grooves in each serpentine microchannel. Due to the distinct hydrodynamic effects induced by different groove orientations, the modified microchannels are categorized into three structural configurations: Structure 1, Structure 2, and Structure 3. Structure 1: The grooves are aligned such that their orientation perfectly coincides with the direction of the centrifugal force acting on the fluid. Simulations were conducted based on Structure 1 to assess the mixing performance of grooves with varying tilt angles. The simulation results revealed that the mixing performance improved as the tilt angle increased. However, when the tilt angle exceeded 45°, the arrangement of grooves became increasingly difficult. Consequently, a tilt angle of 45° was selected for Structure 2. When the grooves are tilted in the opposite direction, the mixing performance is nearly identical to that of Structure 1. For the purpose of better comparative analysis, the tilt angle for Structure 3 was also set to 45°. Structure 2: The grooves in this configuration are rotated 45° relative to Structure 1, which facilitates the predominant entry of fluid along the outer wall into the grooves. Structure 3: Similar to Structure 2, the grooves are rotated by 45°, but the orientation is adjusted to favor fluid flow from the inner wall into the grooves. Figure 3 shows the three-dimensional structure of Structure 2.

### 2.2. Governing Equation

In this study, a single-phase, incompressible, and steady-state laminar flow within a microchannel was analyzed by solving the mass and momentum conservation equations. The continuity equation (Equation (1)) and the steady Navier–Stokes equation (Equation (2)) were employed to describe the mass and momentum conservation, respectively. Additionally, the mass transport equation was solved using the diffusion–convection equation (Equation (3)).

(1) $$\nabla \cdot \vec{v}=0$$

(2) $$\rho \left(\vec{v}\cdot \nabla \right)\vec{v}=-\nabla P+\mu \nabla^{2}\vec{v}$$

(3) $$D\nabla^{2}c-\vec{v}\cdot \nabla c=0$$

where $\vec{v}$ is the velocity vector (m·s^−1^), ρ is the density of fluid (kg·m^−3^), *μ* is the dynamic viscosity of fluid (kg·m^−1^·s^−1^), *P* is the pressure (Pa), *c* is the concentration of the species (mol·m^−3^), and *D* is the diffusion coefficient (m^2^·s^−1^).

The two inlets are specified with a velocity-inlet boundary condition, while the outlet is assigned a pressure-outlet condition with a static pressure of 0. A non-slip boundary condition is applied to the walls. Heat transfer is not considered in this study.

### 2.3. Numerical Solution

In this study, Equations (1)–(3) were numerically solved using ANSYS Fluent 16.0. The Species Transport model was employed to estimate mass fraction distribution within the microchannel. The pressure-based coupled algorithm (Coupled) was employed to implement the coupling between pressure and velocity. The second-order upwind scheme was used for spatial discretization, and the residuals were reduced to less than 10^−6^ to ensure solution convergence.

Water and a dilute fluorescent dye solution were used as the working fluids [41], with identical physical properties: a density of 998 kg·m^−3^, a dynamic viscosity of 1 × 10^−3^ kg·m^−1^·s^−1^, and a diffusion coefficient of 2.3 × 10^−9^ m^2^·s^−1^ [42].

The Reynolds number (*Re*) at the outlet was employed to characterize the fluid flow, which is defined as Equation (4):

(4) Re=Dhuρμ

where *u* is the average velocity of flow (m·s^−1^), *D_h_* is the hydraulic diameter of microchannel outlet (3 × 10^−4^ m), ρ is the density of fluids (998 kg·m^−3^), and *μ* is the dynamic viscosity of fluids (1 × 10^−3^ kg·m^−1^·s^−1^).

The scale and intensity of the segregation can be used to quantitatively assess the mixing degree of the two miscible fluids, which can be determined through statistical methods [43]. The mixing performance of the microchannel is evaluated by the variance of the mass concentration fraction, and it is quantitatively expressed using the mixing index (α), as defined by Equation (6) [44,45]:

(5) $$\sigma =\sqrt{\frac{1}{N}{\sum_{i=1}^{N}\left(c_{i}-{\bar{c}}_{m}\right)}^{2}}$$

(6) $$\alpha =1-\frac{\sigma }{\sigma_{\max}}$$

where σ is the standard deviation of species concentration in the cross-section, *N* is the number of grids within the cross-section, *c_i_* is the mass fraction at point i, ${\bar{c}}_{m}$ represents the average concentration of all sampling points, and $\sigma_{\max}$ is the maximum standard deviation corresponding to completely unmixed fluids. The mixing index (*α)* ranges from 0 (indicating fully separated fluid streams) to 1 (indicating complete mixing).

### 2.4. Grid Independence Verification and Validation of Numerical Simulation

**Figure 4:**
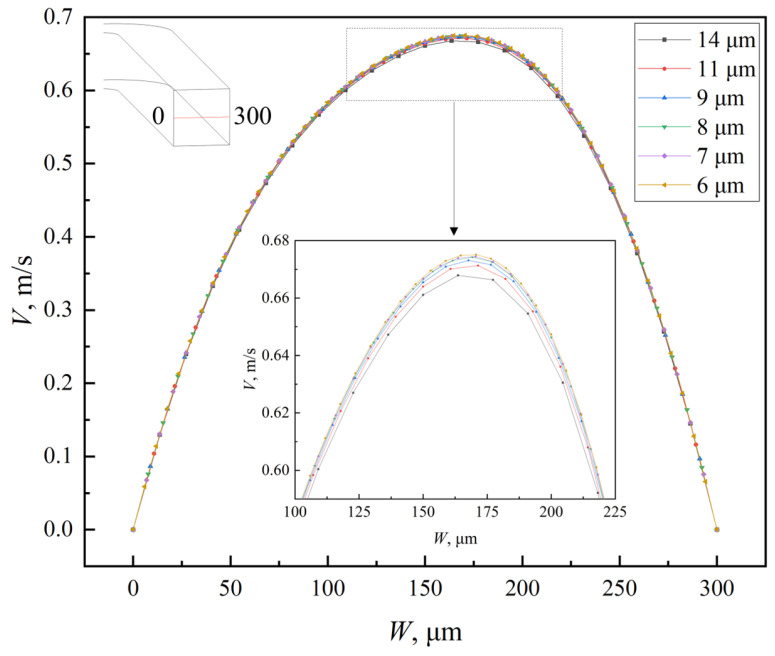
Velocity magnitude distribution of the centerline in outlet cross-section of serpentine microchannel at *Re* = 100.

**Figure 5:**
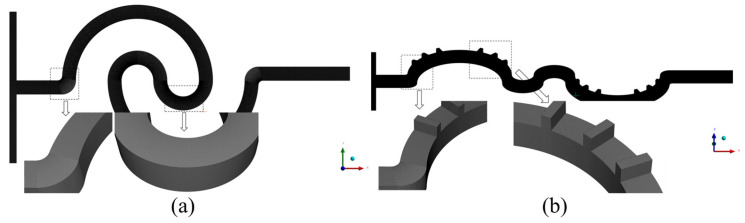
Grid division of microchannels: (**a**) double-spiral and (**b**) serpentine with grooves.

In numerical simulations, the accuracy of the results is highly sensitive to grid density and quality. To enhance the accuracy and optimize computational resources, the grid independence of the serpentine microchannel was verified to determine the optimal cell size. In this study, the microchannels were discretized using hexahedral cells, and six different cell sizes (6, 7, 8, 9, 11, and 14 μm) were tested to assess grid independence. Both the Navier–Stokes and diffusion–convection equations were considered in the analysis. The grid independence verification results are summarized in Table 2, and the velocity distribution along the centerline of the outlet cross-section is shown in Figure 4. As the *Re* increases, the influence of cell size on mixing efficiency becomes more pronounced. This is attributed to the increased number and complexity of Dean vortices at higher *Re*, which require finer grids for accurate representation. For cell sizes of 6 μm and 7 μm, the difference in the mixing index was negligible, with relative errors remaining below 1.5% across all *Re* values. Additionally, the velocity deviation between the two cell sizes is less than 0.1%. Consequently, a uniform cell size of 7 μm was selected for all numerical simulations in this study, as it offers a suitable balance between computational accuracy and efficiency. The grid division of the double-spiral and serpentine microchannel with grooves is shown in Figure 5.

**Figure 6:**
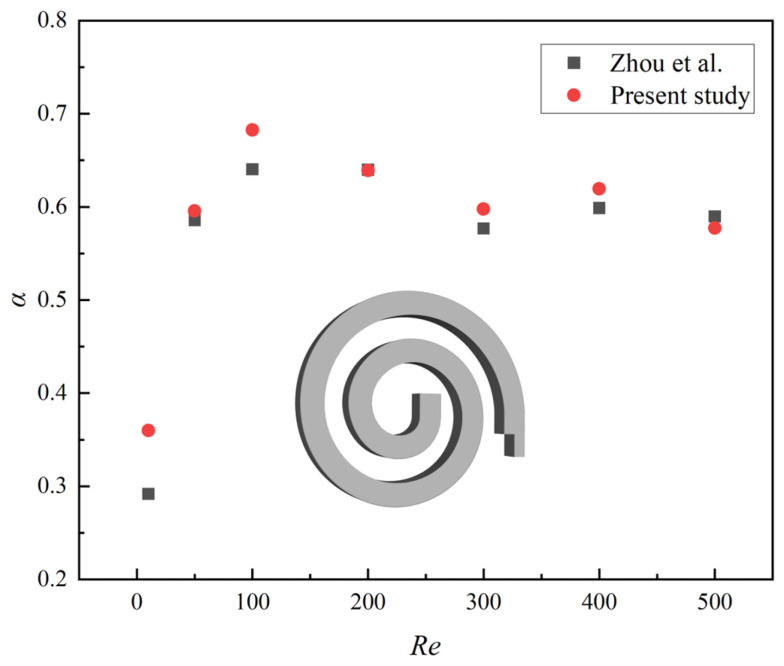
Comparison of the mixing index obtained by numerical simulation in present study and Zhou’s experiments at different *Re*.

The validation of numerical simulation was divided into two parts, with the first part focusing on the validation of the numerical model for the spiral microchannel. A quantitative comparison was conducted with previous work from our research group [21], in which the mixing index of the single-spiral microchannel was compared at different *Re*, as shown in Figure 6. The results demonstrate that our simulation outcomes closely match the experimental data from Zhou et al. [21], with both showing similar trends in the mixing index across a wide range of *Re*. The relative error is less than 5%, except for *Re* = 10, thereby confirming the validity of the numerical simulation. For the validation of the numerical model for a microchannel with grooves, please refer to our research group’s previous work [46]. The modeling process of the serpentine microchannel with grooves in this manuscript, along with the physical models and the numerical methods, is entirely consistent with our previous work [21,46]. Therefore, the numerical simulation results of the serpentine microchannel with grooves are reliable.

## 3. Results and Discussion

### 3.1. Mixing Characteristics of Double-Spiral and Serpentine Microchannels

#### 3.1.1. Concentration Distribution

**Figure 7:**
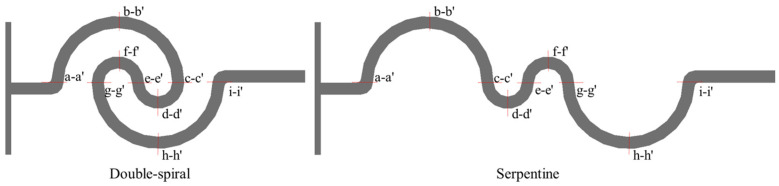
Cross-sectional setup of double-spiral and serpentine microchannels.

To investigate the mechanism of vortex-induced fluid flow and mixing characteristics in the microchannel, nine key cross-sections were set at critical positions within the microchannel, labeled as a-a′ to i-i′, as shown in Figure 7.

**Figure 8:**
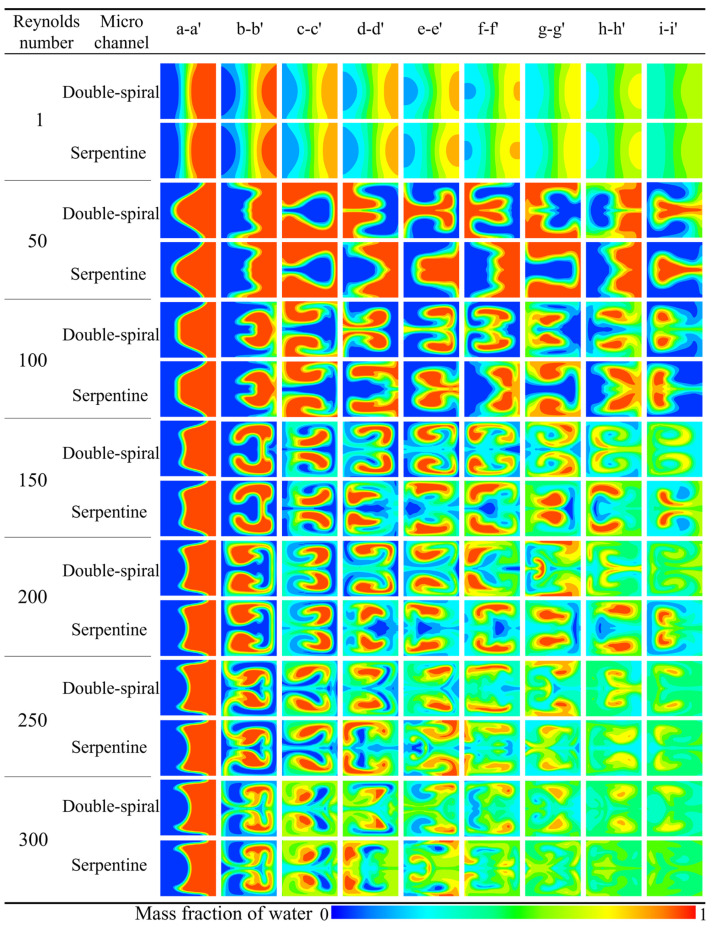
Comparison of concentration distribution at different cross sections in microchannels at different *Re*.

The simulation results for the concentration distribution across nine cross-sections along the microchannel are presented in Figure 8, where blue and red represent the mass fraction of water at 0 and 1, respectively. The double-spiral microchannel exhibits flow reversal only at the e-e′ cross-section, whereas the serpentine microchannel experiences flow reversal at three cross-sections: c-c′, e-e′, and g-g′. These flow reversals are clearly in the concentration distributions. At low *Re*, the flow direction remains consistent up to the e-e′ cross-section in the double-spiral microchannel, causing the contact surface between the fluids to continuously rotate, curl, and stretch in the same direction. This process effectively increases the contact surface area between the two fluids. In contrast, flow reversal compresses the contact surface, thereby limiting the effective utilization of centrifugal forces to enhance fluid mixing. This behavior is particularly evident in the serpentine microchannel, which experiences frequent flow reversals. For *Re* < 200, notable differences in the outlet concentration distributions are observed between the two microchannels. The double-spiral microchannel exhibits superior color uniformity compared to the serpentine microchannel. However, when *Re* exceeds 200, the folding and swirling motions of the fluids intensify significantly, leading to enhanced mixing. In the high *Re* regime, the serpentine microchannel shows better color uniformity than the double-spiral microchannel, in contrast to the lower *Re* conditions.

#### 3.1.2. Mixing Performance

**Figure 9:**
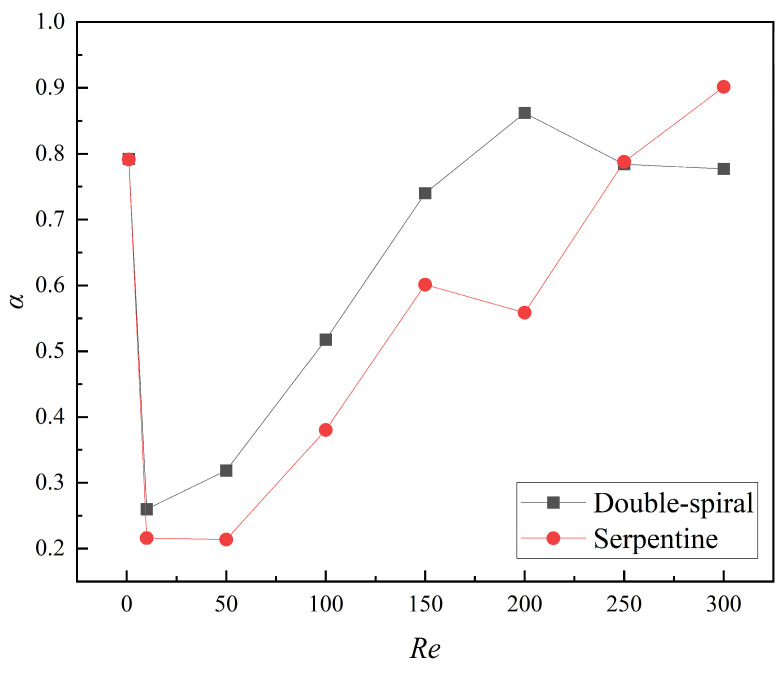
Variation of mixing index at the outlet of the microchannels with *Re* from 1 to 300.

The numerical simulation results for the mixing index at the outlet of the microchannels across a *Re* range of 1 to 300 are shown in Figure 9. At *Re* = 1, the mixing index is notably high due to the long residence time of the fluid at such a low flow rate, where molecular diffusion predominates and facilitates efficient mixing. When *Re* is less than 10, a sharp decline in the mixing index is observed. This is due to a significant reduction in residence time and insufficient intensity of the Dean vortices, which fail to induce substantial flow disturbance. As *Re* continues to increase, the intensity of the Dean vortices gradually intensifies, exerting a stronger influence on the flow characteristics, which results in a corresponding increase in the mixing index for both microchannels. In the range of *Re* = 150–200, the mixing index for the double-spiral microchannel continues to increase, whereas the mixing index for the serpentine microchannel begins to decrease. For *Re* < 200, the mixing index of the double-spiral microchannel consistently exceeds that of the serpentine microchannel. However, a notable transition occurs at *Re* = 250–300, where the mixing performance of the double-spiral microchannel deteriorates, while the serpentine microchannel experiences a significant improvement in mixing efficiency. Ultimately, the serpentine microchannel surpasses the double-spiral microchannel in terms of mixing efficiency at higher *Re*.

#### 3.1.3. Dean Vortex and Intensity

**Figure 10:**
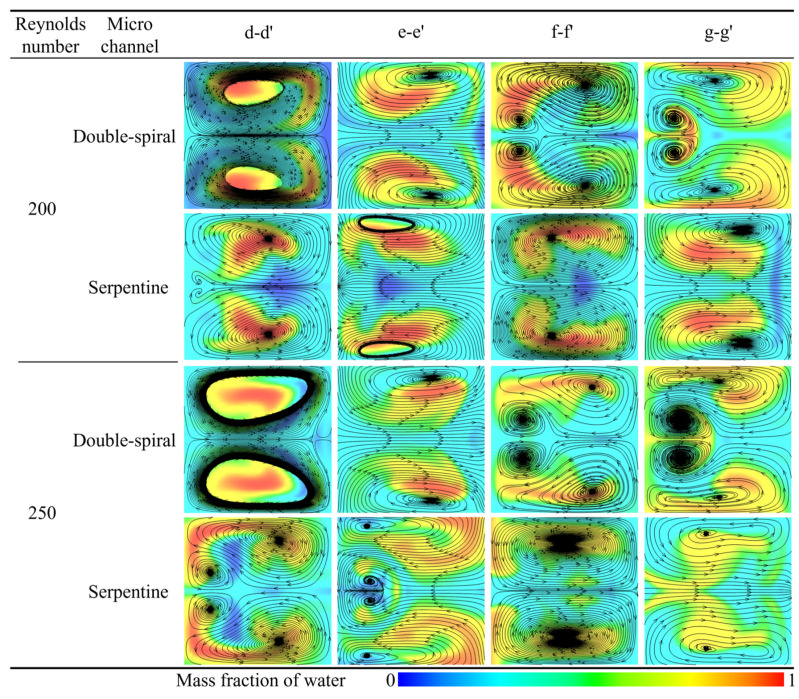
Comparison of streamtraces at different cross sections in the two microchannels.

The flow direction and morphological evolution of Dean vortices at the cross-sections are clearly depicted through the streamlines shown in Figure 10. In this figure, blue and red represent the mass fraction of water at 0 and 1, respectively. For *Re* < 200, both microchannels display a single pair of vortices at the cross-sections. However, at Re = 200, the Dean vortices in the double-spiral microchannel evolve from a single pair into two pairs at the f-f′ cross-section, driven by centrifugal force reversal. In the serpentine microchannel, a new pair of vortices initially appears at the d-d′ cross-section, but disappears after the centrifugal force reversal at the e-e′ cross-section, without reappearing thereafter. These distinct vortex evolution patterns help explain the contrasting trends observed in the mixing indices between the two microchannel geometries. In the double-spiral microchannel, the formation of a four-vortex structure leads to a continuous increase in the mixing index, as shown in Figure 9. In contrast, the serpentine microchannel, due to the absence of significant changes in vortex structure, experiences a decline in the mixing index. As *Re* increases, the intensity of the vortices increases. In the serpentine microchannel, the newly formed pair of Dean vortices fully develops at the d-d′ cross-section, extending through the e-e′ cross-section at *Re* = 250. Compared to *Re* = 200, this new pair of Dean vortices significantly enhances the stretching effect on the contact surface between the fluids, resulting in an increased contact surface area, as shown in Figure 8, which improves mixing efficiency. At *Re* = 250, the serpentine microchannel induces the four-vortex structure earlier, leading to a stronger stretching effect and a substantial increase in the mixing index, as shown in Figure 9. In contrast, the vortex structure in the double-spiral microchannel exhibits minimal change compared to *Re* = 200, and the reduced residence time results in a decrease in the mixing index. This shift in vortex behavior contributes to the reversal in the mixing index trends between the two microchannels. These observations suggest that the reversal of centrifugal force direction is directly linked to the formation patterns of vortices, which influence both the intensity and efficiency of vortex-induced mixing.

Numerous methods have been developed for the quantitative characterization of vortex intensity, with the swirling strength proposed by Zhou and Adrian [47,48] offering significant advantages. Zhou et al. introduced a framework in which, in three-dimensional flow conditions, the local velocity gradient tensor has one real eigenvalue (*λ*_r_) and a pair of complex conjugate eigenvalues (*λ*_cr_ ± i*λ*_ci_) when the discriminant of its characteristic equation is positive. The local flow in the plane spanned by the two complex eigenvectors corresponding to these complex eigenvalues exhibits rotational motion. The intensity of this local rotational motion can be quantified by the imaginary part of the complex eigenvalue, *λ*_ci_. Thus, they defined the imaginary component, *λ*_ci_, as the swirling strength of the vortex. This approach effectively identifies vortices and quantifies their intensity. The fundamental advantage of this method lies in its ability to isolate the rigid rotational motion of fluid elements, eliminating the influence of shear deformations. This results in precise localization of the vortex core and enables direct quantification of vortex intensity.

**Figure 11:**
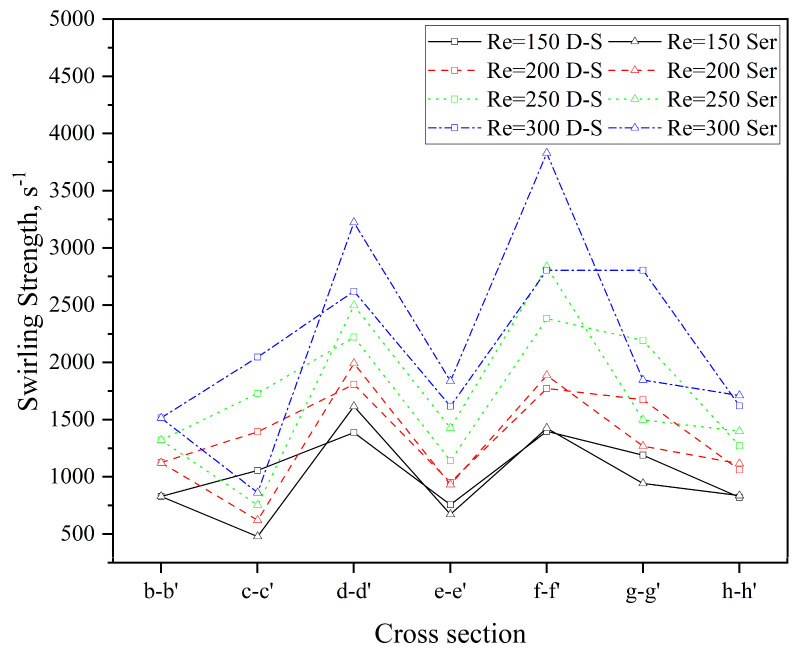
Comparison of swirling strength at different cross-sections in the two microchannels.

In this study, swirling strength is employed to conduct a quantitative comparative analysis of the intensity of Dean vortices between the two microchannels. The variation in swirling strength across different cross-sections in both microchannels at various *Re* is presented in Figure 11. As observed, the swirling strength at each cross-section gradually increases with *Re*. At a given *Re*, however, the swirling strength of the double-spiral (D-S) and serpentine (Ser) microchannels exhibits markedly divergent trends. The reversal of centrifugal force weakens the rotation of the original Dean vortices until the direction of rotation is fully inverted. As the vortices rotate in the opposite direction and collide with one another, the viscosity of the fluid dissipates the organized rotational kinetic energy into disordered thermal energy. This energy dissipation leads to a substantial reduction in swirling strength at the c-c′, e-e′, and g-g′ cross-sections of the serpentine microchannel, compared to earlier cross-sections. In contrast, the swirling strength of the double-spiral microchannel decreases only at the e-e′ cross-section. The reduction in swirling strength at the h-h′ cross-section in both microchannels is attributed to the increase in curvature radii. As shown in Figure 10, for *Re* < 200, the swirling strength in both microchannels is insufficient to generate a second pair of Dean vortices. At *Re* = 200, the reversal of centrifugal force in the double-spiral microchannel enables the formation of a second pair of Dean vortices. In contrast, in the serpentine microchannel, vortex energy is dissipated due to an earlier flow reversal, resulting in only a single pair of Dean vortices. At *Re* = 250, the swirling strength increases, facilitating the formation of a second pair of Dean vortices following the initial centrifugal force reversal in the serpentine microchannel. At *Re* = 300, two pairs of Dean vortices appear at most cross-sections in both microchannels. As *Re* increases, the swirling strength of the serpentine microchannel gradually exceeds that of the double-spiral microchannel from the d-d′ to f-f′ cross-sections. This trend helps explain why the mixing performance of the serpentine microchannel surpasses that of the double-spiral microchannel at higher *Re*, particularly in terms of vortex intensity.

### 3.2. Enhancement of Mixing Performance of Serpentine Microchannel

The mixing index of the serpentine microchannel is relatively low within the *Re* range of 10 to 200. To enhance its mixing performance, grooves with varying angles, as depicted in Figure 2, are incorporated into the microchannel design. The impact of these groove modifications on the mixing characteristics of the serpentine microchannel is subsequently investigated through numerical simulations.

#### 3.2.1. The Effect of Grooves on Concentration Distribution

**Figure 12:**
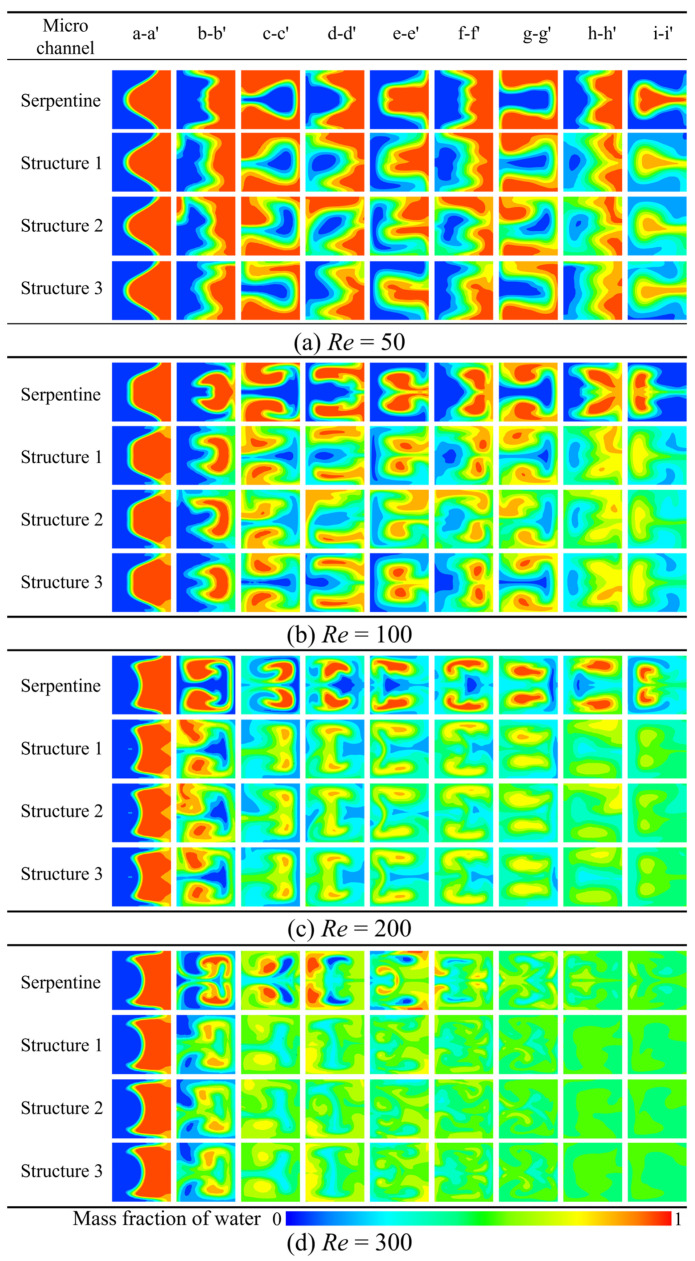
Comparison of concentration distribution at different cross sections: (**a**) *Re* = 50, (**b**) *Re* = 100, (**c**) *Re* = 200, (**d**) *Re* = 300.

The introduction of grooves into the microchannel significantly alters the flow pattern, making it more complex. The concentration distribution of the four microchannels at different *Re* is presented in Figure 12, where blue and red represent the mass fraction of water at 0 and 1, respectively. In the serpentine microchannel without grooves, the flow pattern is initially symmetrical; however, the presence of grooves leads to considerable irregular deformation in the three microchannels with grooves. Under the synergistic effect of the grooves and the Dean vortices, stronger twisting and stretching forces are exerted on the contact surface, thereby enhancing the mixing. As *Re* increases, both the intensity of the Dean vortices and the synergistic enhancement of the mixing become more pronounced. The enhancement of mixing becomes more pronounced when *Re* > 100, as the color uniformity at the d-d′ cross-section of the serpentine microchannels with grooves is significantly higher.

#### 3.2.2. The Effect of Grooves on Mixing Performance

**Figure 13:**
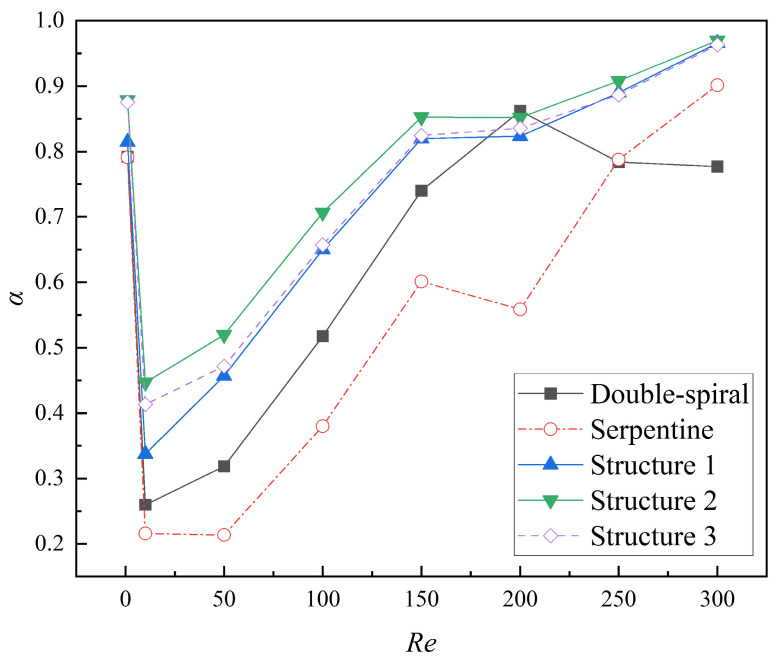
Variation of mixing index at the outlet of microchannels with *Re* from 1 to 300.

The comparison of mixing indices across five microchannels within the *Re* range of 1 to 300 is illustrated in Figure 13. The results demonstrate that the mixing index of the serpentine microchannel with grooves is significantly higher than that of both the serpentine microchannel without grooves and the double-spiral microchannel. This finding is consistent with the analysis of concentration distribution, further confirming the substantial enhancement of mixing performance attributed to the groove structure in the serpentine microchannel. Over the examined *Re* range, the average mixing indices for Structures 1, 2, and 3 are 43.5%, 58.0%, and 50.2% higher, respectively, compared to the serpentine microchannel without grooves, and 18.2%, 29.6%, and 23.7% higher than those of the double-spiral microchannel. These results provide clear evidence that the groove structure with varying angles can effectively enhance mixing performance, with Structure 2 exhibiting the most significant improvement. Notably, the mixing index of Structure 2 remains above 0.5 across the entire *Re* range and surpasses 0.7 for *Re* > 100. The mixing index peaks at 0.97 for *Re* = 300, underscoring its exceptional mixing capability.

#### 3.2.3. The Effect of Grooves on Mixing Cost

**Figure 14:**
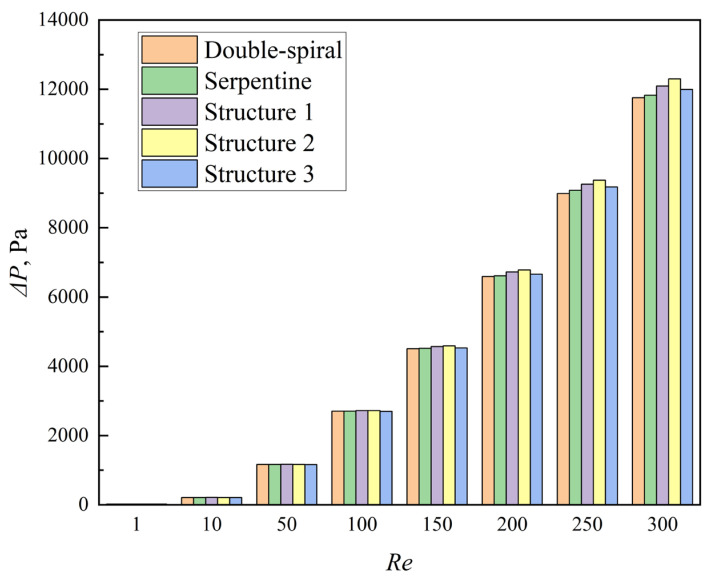
Comparison of pressure drop of the microchannels with *Re* from 1 to 300.

The pressure drop (Δ*P*) across the microchannels is presented in Figure 14. In general, the serpentine microchannel exhibits a slightly higher pressure drop compared to the double-spiral microchannel. The introduction of grooves further increases the pressure drop. Specifically, the average pressure drops for Structures 1, 2, and 3 are 1.0%, 1.4%, and 0.2% higher, respectively, than that of the serpentine microchannel without grooves.

Passive microchannels primarily rely on pump-generated energy to facilitate fluid mixing. However, assessing the performance of microchannels solely in terms of mixing efficiency or pressure drop is inadequate. To provide a more comprehensive evaluation, Chung et al. [49] introduced the concept of mixing cost (*MC*), defined as the ratio of the mixing index to the pressure drop. This metric enables a more thorough evaluation of performance of microchannels across different structural configurations.

(7) $$MC=\frac{\alpha }{\Delta P}$$

where *α* is mixing index, Δ*P* is pressure drop of microchannel. *MC* is particularly useful for comparing microchannels with the same flow rate, as it relates the mixing index to the unit pressure drop. A higher *MC* value indicates that the microchannel achieves a better mixing index for the same energy consumption, thereby reflecting superior overall performance.

**Figure 15:**
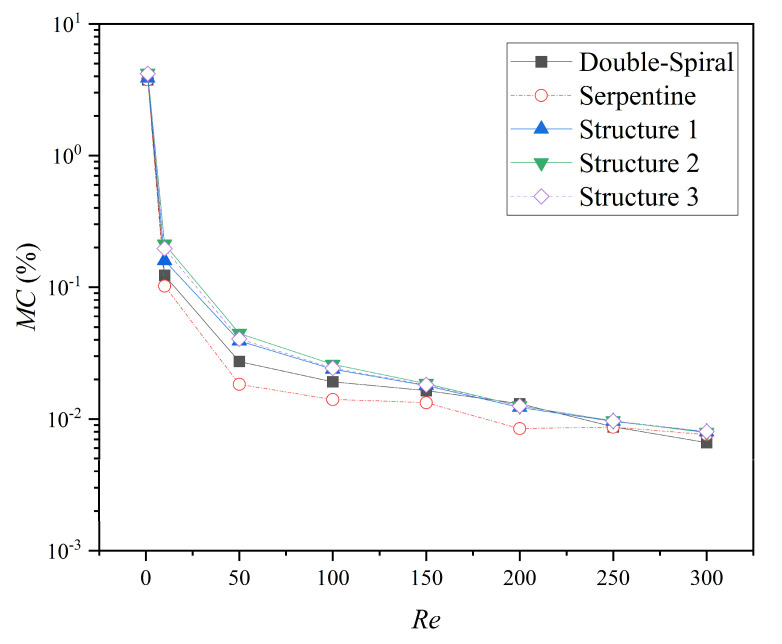
The *MC* values of microchannels at different *Re*.

The comparison of the *MC* for the five microchannels at different *Re* is presented in Figure 15. The *MC* of all microchannels decreases as *Re* increases, following a decelerating trend after an initial rapid decline. For *Re* < 250, the microchannels with grooves exhibit the highest *MC*, followed by the double-spiral microchannel, with the serpentine microchannel showing the lowest *MC*. At *Re* = 300, the double-spiral microchannel exhibits the lowest *MC*, while the *MC* values of the serpentine microchannel and the microchannels with grooves become comparable. Specifically, the *MC* values of Structures 1, 2, and 3 are 42.2%, 56.3%, and 50.0% higher than that of the serpentine microchannel, especially within the *Re* range of 10 to 100, highlighting the enhancement effect of the groove structure on the mixing performance of the serpentine microchannel. Notably, although Structure 2 exhibits the highest pressure drop (Δ*P*), it achieves the highest *MC* value among all microchannels, indicating that Structure 2 achieves the optimal balance between efficient mixing and energy consumption.

#### 3.2.4. The Effect of Grooves on Swirling Strength

**Figure 16:**
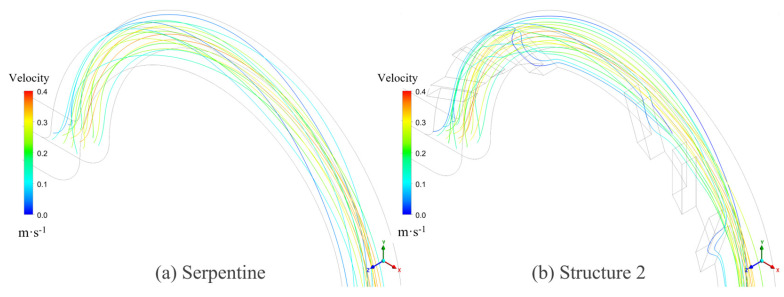
The streamlines of microchannels at *Re* = 50: (**a**) serpentine and (**b**) Structure 2.

**Figure 17:**
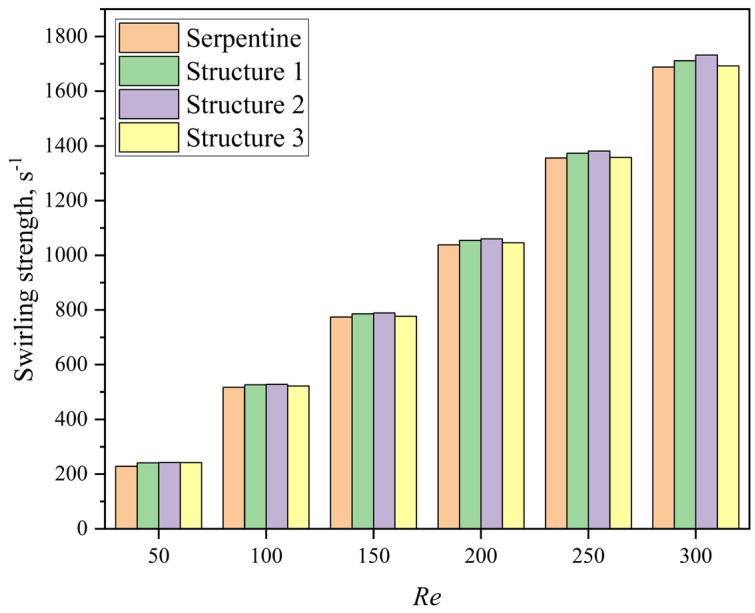
The swirling strength values of microchannels at different *Re*.

On one hand, the groove structure increases the intensity of vortices within the microchannel; on the other hand, it disrupts the symmetry of the Dean vortices, leading to a shift in their position along the channel, which further enhances mixing. As shown in Figure 16, the streamline patterns at *Re* = 50 for the serpentine and Structure 2 microchannels reveal that the grooves induce lateral flow, thereby increasing the vortex intensity and leading to vortex displacement. The comparison of the volume-averaged swirling strength in the channel at different *Re* is presented in Figure 17. The swirling strength in microchannels with grooves is higher than that in the serpentine microchannel, with Structure 2 exhibiting the highest value. The secondary flow induced by the grooves in Structure 2 aligns with the secondary flow direction of the Dean vortices, as depicted in Figure 16b. The interaction and superposition of these two flow components result in a significantly higher vortex intensity within the channel for Structure 2 compared to Structures 1 and 3. This indicates that the vortex modifications induced by the grooves in Structure 2 are particularly effective in enhancing mixing performance. These results are in agreement with the findings of Yuan et al. [50], who demonstrated that increased rotational strength can enhance mass transfer.

**Figure 18:**
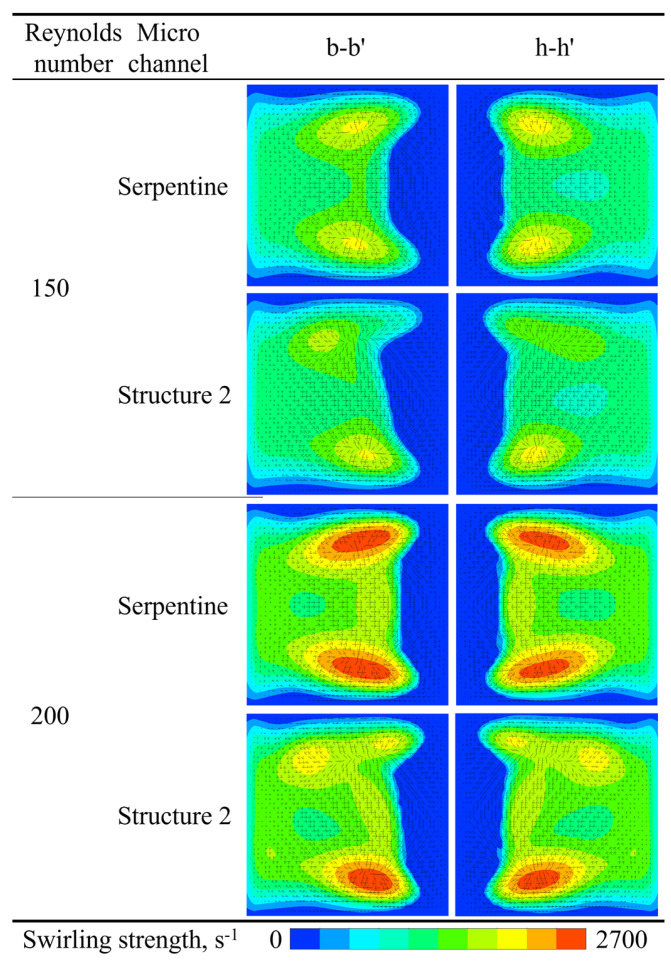
Comparison of the swirling strength contour and velocity vectors of microchannels.

The swirling strength contours and velocity vectors at the b-b′ and h-h′ cross-sections of the serpentine microchannel and Structure 2 are compared to illustrate the effect of grooves on vortex dynamics, as shown in Figure 18. At the b-b′ cross-section, two symmetrical Dean vortices are observed in the serpentine microchannel. In contrast, the position of maximum vortex intensity shifts in Structure 2, causing the originally symmetrical vortex structure to deflect to the right. Consequently, the center of the velocity vectors also shifts. The differences become more pronounced at the h-h′ cross-section. The symmetrical swirling strength contour observed in the serpentine microchannel becomes asymmetric in Structure 2, with one side exhibiting significantly higher strength than the other. This asymmetry in vortex strength results in an uneven flow pattern, further enhancing mixing.

## 4. Conclusions

This study investigates the mixing characteristics of double-spiral and serpentine microchannels through numerical simulations over a *Re* range of 1 to 300. The results indicate that, for *Re* < 200, the mixing index of the double-spiral microchannel is 36.6% higher than that of the serpentine microchannel. This discrepancy is attributed to the more efficient vortex utilization in the double-spiral configuration, as the serpentine microchannel undergoes three centrifugal force reversals, which dissipate vortex energy and reduce swirling strength. At *Re* = 200, the double-spiral microchannel generates two pairs of Dean vortices, significantly improving its mixing efficiency, while the serpentine microchannel has only a single pair of vortices, resulting in lower mixing performance. However, a critical transition occurs at *Re* = 250–300, where the serpentine microchannel forms two pairs of Dean vortices at an earlier stage, significantly enhancing the stretching effect on the contact surface between the fluids. This early formation of four-vortex structures leads to the serpentine microchannel outperforming the double-spiral microchannel in terms of mixing efficiency.

The introduction of grooves significantly improves the mixing performance of the serpentine microchannel by inducing asymmetric vortices and enhancing the vortex intensity. Consequently, the mixing indices for Structures 1, 2, and 3 are 43.5%, 58.0%, and 50.2% higher, respectively, compared to the serpentine microchannel without grooves. Furthermore, the average mixing cost (*MC*) for Structures 1, 2, and 3 is 42.2%, 56.3%, and 50.0% higher, respectively, than that of the serpentine microchannel. These results underscore the substantial optimization effects of the groove structure on the mixing characteristics of the serpentine microchannel. Notably, Structure 2 exhibits the highest mixing efficiency and *MC*, as the secondary flow induced by the grooves aligns with the secondary flow direction of the Dean vortices. The interaction and superposition of these two flow components result in a significant increase in vortex intensity.

This study is based on a steady-state analysis, and further research into unsteady processes is necessary to fully understand the dynamics of the mixing behavior. Additionally, future work should include experimental validation and scale-up studies to meet the requirements for industrial applications.

## Figures and Tables

**Table 1:** Geometric parameters of microchannels.

Geometric Parameters	Value	Unit
Inlet 1 width (*W*_1_)	150	μm
Inlet 2 width (*W*_2_)	150	μm
Outlet and channel width (*W*)	300	μm
Length of inlet channel (*L*_0_)	3.3	mm
Length of Inlet 1 and Inlet 2 channel (*L*_1_)	1.5	mm
Length of straight channel (*L*_2_)	1.0	mm
Length of outlet channel (*L*_3_)	2.0	mm
Length of mixing channel	15.996	mm
Radius of big semicircle (*R*_1_)	1.5	mm
Radius of small semicircle (*R*_2_)	0.5	mm

**Table 2:** The mixing index and relative error at different cell sizes.

Cell Size μm	Number of Grids	*Re* = 1		*Re* = 10		*Re* = 100		*Re* = 300	
		*α*	Error	*α*	Error	*α*	Error	*α*	Error
14	572,418	0.7911	−0.03%	0.2328	8.31%	0.5600	48.59%	0.9555	7.54%
11	1,208,844	0.7912	−0.02%	0.2241	4.29%	0.4766	26.47%	0.9387	5.65%
9	2,193,510	0.7912	−0.01%	0.2197	2.21%	0.4267	13.23%	0.9255	4.16%
8	3,246,606	0.7913	−0.01%	0.2179	1.36%	0.4206	11.60%	0.9151	2.99%
7	4,732,408	0.7913	0.00%	0.2160	0.51%	0.3802	0.88%	0.9015	1.47%
6	7,459,000	0.7913	—	0.2149	—	0.3769	—	0.8885	—

## Data Availability

The original contributions presented in this study are included in the article. Further inquiries can be directed to the corresponding author.
